# Supplementary figures and images for: Association between depression during pregnancy and preterm birth: Results from population cohorts and mouse experimental models
Source: PLoS One. 2026 Jan 29;21(1):e0341449. doi: 10.1371/journal.pone.0341449 (PMC12854446; doi:10.1371/journal.pone.0341449)

**sFigure1**.Comparison of birth numbers of mice in the CUMS group and the control group


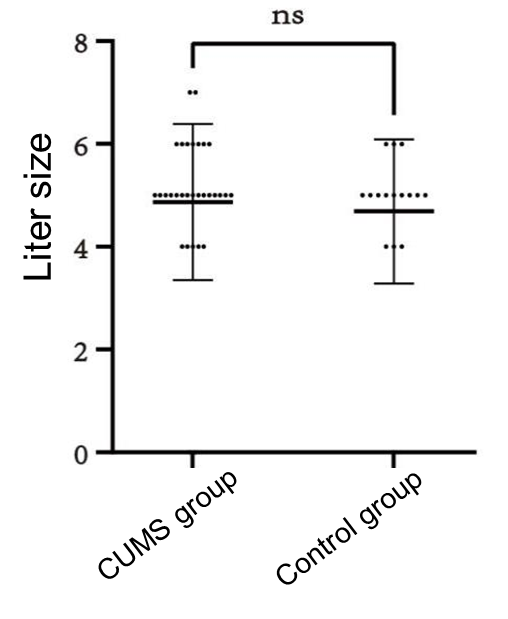

Supplement: S1 Fig — (DOC) [file pone.0341449.s001.doc]
