# Supplementary material for: Association between depression during pregnancy and preterm birth: Results from population cohorts and mouse experimental models
Source: PLoS One. 2026 Jan 29;21(1):e0341449. doi: 10.1371/journal.pone.0341449 (PMC12854446; doi:10.1371/journal.pone.0341449)
Supplement: S1 Table — (DOC) [file pone.0341449.s002.doc]

**sTable1.**Univariate and Multivariate Analysis of Pregnant with Premature Birth

| Variable | Univariate Analysis | |  | Multivariate Analysis | |
| --- | --- | --- | --- | --- | --- |
|  | RR(95%CI) | *P* value |  | RR(95%CI) | *P* value |
| Age (years) |  |  |  |  |  |
| <25 | Ref |  |  | Ref |  |
| 25~34 | 0.56(0.31,1.02) | 0.057 |  | 0.74(0.39,1.40) | 0.354 |
| ≥35 | 0.64(0.25,1.60) | 0.337 |  | 0.91(0.35,2.40) | 0.851 |
| Minority |  |  |  |  |  |
| Yes | Ref |  |  | Ref |  |
| No | 1.05(0.44,2.47) | 0.916 |  | 0.94(0.39,2.26) | 0.885 |
| Residence |  |  |  |  |  |
| Town | Ref |  |  | Ref |  |
| Rural | 1.61(1.00,2.60) | 0.05 |  | 1.27(0.75,2.14) | 0.374 |
| Marital status |  |  |  |  |  |
| Married/cohabitatinge | Ref |  |  | Ref |  |
| Single/divorced | 2.25(0.78,6.52) | 0.134 |  | 1.97(0.65,5.92) | 0.228 |
| Education level |  |  |  |  |  |
| At least primary school level | Ref |  |  | Ref |  |
| At least secondary school level | 0.67(0.34,1.31) | 0.246 |  | 0.74(0.37,1.48) | 0.395 |
| Advanced level and tertiary level | 0.47(0.26,0.86) | 0.014 |  | 0.59(0.31,1.15) | 0.123 |
| Occupation |  |  |  |  |  |
| Employed, | Ref |  |  | Ref |  |
| Not employed | 0.96(0.50,1.85) | 0.899 |  | 0.84(0.42,1.65) | 0.605 |
| Average household income |  |  |  |  |  |
| ≤$16,500 | Ref |  |  | Ref |  |
| ＞$16,500 | 0.49(0.21,1.15) | 0.100 |  | 0.64(0.26,1.53) | 0.314 |
| Pregnant (weeks) |  |  |  |  |  |
| Early pregnancy（<14 ) | Ref |  |  | Ref |  |
| Mid-pregnancy (14 ~28 ) | 1.19(0.61 2.36) | 0.608 |  | 1.13(0.56,2.25) | 0.736 |
| Late pregnancy (≥28 ) | 0.43(0.22,0.84) | 0.014 |  | 0.41(0.21,0.81) | 0.010 |

Univariate analysis: no adjustment for confounding factors;

Multivariate analysis: adjust age, nationality, residence, marital status, education level, occupation status, annual family income, pregnancy.
