# Supplementary material for: Association between depression during pregnancy and preterm birth: Results from population cohorts and mouse experimental models
Source: PLoS One. 2026 Jan 29;21(1):e0341449. doi: 10.1371/journal.pone.0341449 (PMC12854446; doi:10.1371/journal.pone.0341449)
Supplement: S2 Table — (DOC) [file pone.0341449.s003.doc]

**sTable2.** Behavioral changes in the CUMS group (x±s)

| Variable | Week 2 | Week 4 | Week 6 | *P* value |
| --- | --- | --- | --- | --- |
| Body Mass (g) | 17.10±0.83 | 17.96±1.01 | 19.69±0.90 | 0.352 |
| SPT |  |  |  |  |
| preference rate (%) | 0.92±0.04 | 0.88±0.06 | 0.77±0.07 | 0.026 |
| OFT |  |  |  |  |
| Distance (cm) | 515.5±319.51 | 613.±312.96 | 460.52±149.31 | 0.019 |
| Central time(s) | 22.09±10.27 | 17.36±10.05 | 11.98±7.27 | 0.019 |
| Central /Total (%) | 23.26±10.04 | 18.79±8.23 | 15.17±5.94 | <0.001 |
| FST |  |  |  |  |
| Static time(s) | 49.30±23.58 | 103.3±70.90 | 134.40±67.24 | <0.001 |
| TST |  |  |  |  |
| Static time(s) | 23.60±8.98 | 46.60±25.60 | 67.20±34.85 | 0.026 |
