# Supplementary material for: Association between depression during pregnancy and preterm birth: Results from population cohorts and mouse experimental models
Source: PLoS One. 2026 Jan 29;21(1):e0341449. doi: 10.1371/journal.pone.0341449 (PMC12854446; doi:10.1371/journal.pone.0341449)
Supplement: S3 Table — (DOC) [file pone.0341449.s004.doc]

**sTable3** Pregnancy between CUMS and control group

| Conception status | All  (n=80) | Control group  (n=20) | CUMS group  (n=60) | χ^2^ | *P* value |
| --- | --- | --- | --- | --- | --- |
| First conception |  |  |  | 3.75 | 0.053 |
| Yes | 41 (51.25) | 14(34.15) | 27(65.85) |  |  |
| No | 39 (48.75) | 6 (15.38) | 33(84.62) |  |  |
| Total conception |  |  |  | 4.44 | 0.035 |
| Yes | 48(60.00) | 16(33.33) | 32(66.67) |  |  |
| No | 32(40.00) | 4(12.50) | 28 (87.50) |  |  |
