# Supplementary material for: Association between depression during pregnancy and preterm birth: Results from population cohorts and mouse experimental models
Source: PLoS One. 2026 Jan 29;21(1):e0341449. doi: 10.1371/journal.pone.0341449 (PMC12854446; doi:10.1371/journal.pone.0341449)
Supplement: S4 Table — (DOC) [file pone.0341449.s005.doc]

**sTable4.Weight in CUMS group and control group**

| Variable | Weight gain during pregnancy (g) | |  | Neonatal rat body weight (1 day, g) | |
| --- | --- | --- | --- | --- | --- |
|  | n=48 | mean ±SD |  | n=177 | mean±SD |
| Control group | 16（33.33） | 10.05±1.74 |  | 57（32.20） | 1.28±0.15 |
| CUMS group | 32（66.67） | 10.48±2.27 |  | 120（67.80） | 1.33±0.15 |
