# Supplementary material for: Association between depression during pregnancy and preterm birth: Results from population cohorts and mouse experimental models
Source: PLoS One. 2026 Jan 29;21(1):e0341449. doi: 10.1371/journal.pone.0341449 (PMC12854446; doi:10.1371/journal.pone.0341449)
Supplement: Attachment — Submitted filename: Response to reviewers .doc [file pone.0341449.s011.doc]

# Author response to academic editor and reviewers

MANUSCRIPT ID: PONE-D-24-60622

MANUSCRIPT TITLE: Association between depression during pregnancy and preterm birth: results from

population cohorts and mouse experimental models

| **academic editor** | **AUTHOR RESPONSE** | **PAGE NUMBER** |
| --- | --- | --- |
| （1）Please ensure that your manuscript meets PLOS ONE's style requirements, including those for file naming. The PLOS ONE style templates can be found at  https://journals.plos.org/plosone/s/file?id=wjVg/PLOSOne_formatting_sample_main_body.pdf and  [**https://journals.plos.org/plosone/s/file?id=ba62/PLOSOne_formatting_sample_title_authors_affiliations.pdf**](https://journals.plos.org/plosone/s/file?id=ba62/PLOSOne_formatting_sample_title_authors_affiliations.pdf) | Thank you for your guidance regarding PLOS ONE's style requirements. We have carefully reviewed the journal's templates and made the adjustments to ensure full compliance. We confirm that the revised manuscript has been checked against both provided templates and complies with all PLOS ONE style requirements. The track-changes version highlights all modifications made.  Thank you for your careful review and for the opportunity to improve our manuscript | - |
| (2) To comply with PLOS ONE submissions requirements, in your Methods section, please provide additional information regarding the experiments involving animals and ensure you have included details on (1) methods of sacrifice, (2) methods of anesthesia and/or analgesia, and (3) efforts to alleviate suffering. | Thank you for your important request regarding animal experiment details. We have expanded the Methods section to include all required information, with the following specific additions  **At the conclusion of the study, mice were euthanized via cervical dislocation under isoflurane anesthesia. No invasive procedures requiring analgesia were performed during behavioral tests. Efforts to minimize suffering included daily health monitoring, environmental enrichment (nesting materials), and immediate intervention for signs of severe distress (none observed)**  The track-changes version highlights all modifications. We appreciate your rigorous standards and hope these clarifications address all concerns. | Line 19-23 on page 9. |
| (3) We note that the grant information you provided in the ‘Funding Information’ and ‘Financial Disclosure’ sections do not match. When you resubmit, please ensure that you provide the correct grant numbers for the awards you received for your study in the ‘Funding Information’ section. | Thank you for bringing this important discrepancy to our attention. We sincerely apologize for the oversight in our original submission. We have now carefully verified and standardized all funding information as follows:  This study was supported by National Natural Science Foun-dation of China (81960592,82073569,82260645), Basic Research of Yunnan Province-Key Project (202101AS070040), Basic Research of Yunnan Province-Excellent Youth Project (202001AW070021), Spe-cial Project for Selection of High-level Scientific and Technological Talents and Innovation Team-Reserve Talent Program for Young and Middle-aged Academic and Technical Leaders (202005AC160023), Basic Research of Yunnan Province Joint Special Project of Kunming Medical University (202101AY070001), Doctoral Research Fund Pro-ject of the First Afiliated Hospital of Kunming Medical University (2022BS013).  We appreciate your careful review and the opportunity to correct this administrative detail. Please don't hesitate to contact us if any additional clarification regarding funding would be helpful. | Line 16-23on page 1. |
| （4）Thank you for stating the following financial disclosure: This project is supported in part by a Kunming Medical University grant.  Please state what role the funders took in the study. If the funders had no role, please state: "The funders had no role in study design, data collection and analysis, decision to publish, or preparation of the manuscript." If this statement is not correct you must amend it as needed. Please include this amended Role of Funder statement in your cover letter; we will change the online submission form on your behalf. | Thank you for your guidance regarding the funder’s role statement. We confirm that Kunming Medical University, as the funding body, had no involvement in any aspect of the research process. We have made the following updates at cover letter: Portions of this study were supported by Kunming Medical University [202101AY070001, 2022BS013]. Funders play a role in data collection and analysis, publication decisions, or manuscript preparation.  We appreciate your attention to research integrity matters and confirm full compliance with PLOS ONE's funding disclosure policies. Please don't hesitate to contact us if any additional information would be helpful. | Cover letter  Line 14-16  on page 1. |
| （5）Thank you for stating the following in the Competing Interests section: no Please confirm that this does not alter your adherence to all PLOS ONE policies on sharing data and materials, by including the following statement: "This does not alter our adherence to PLOS ONE policies on sharing data and materials.” (as detailed online in our guide for authors http://journals.plos.org/plosone/s/competing-interests). If there are restrictions on sharing of data and/or materials, please state these. Please note that we cannot proceed with consideration of your article until this information has been declared. Please include your updated Competing Interests statement in your cover letter; we will change the online submission form on your behalf. | Thank you for your guidance regarding PLOS ONE's competing interests and data sharing policies. We are pleased to confirm the following: Revised in manuscript and submission system: "The authors declare no competing interests. This does not alter our adherence to PLOS ONE policies on sharing data and materials."  We appreciate your careful attention to these important publication ethics requirements. Please don't hesitate to contact us if any additional clarification would be helpful. | Line 11-12  on page 2. |
| (6)  We note that your Data Availability Statement is currently as follows: no  Please confirm at this time whether or not your submission contains all raw data required to replicate the results of your study. Authors must share the “minimal data set” for their submission. PLOS defines the minimal data set to consist of the data required to replicate all study findings reported in the article, as well as related metadata and methods (https://journals.plos.org/plosone/s/data-availability#loc-minimal-data-set-definition).  For example, authors should submit the following data:  - The values behind the means, standard deviations and other measures reported; - The values used to build graphs; - The points extracted from images for analysis.  Authors do not need to submit their entire data set if only a portion of the data was used in the reported study.  If your submission does not contain these data, please either upload them as Supporting Information files or deposit them to a stable, public repository and provide us with the relevant URLs, DOIs, or accession numbers. For a list of recommended repositories, please see https://journals.plos.org/plosone/s/recommended-repositories.  If there are ethical or legal restrictions on sharing a de-identified data set, please explain them in detail (e.g., data contain potentially sensitive information, data are owned by a third-party organization, etc.) and who has imposed them (e.g., an ethics committee). Please also provide contact information for a data access committee, ethics committee, or other institutional body to which data requests may be sent. If data are owned by a third party, please indicate how others may request data access. | Thank you for your guidance regarding data availability requirements. We have taken the following steps to ensure full compliance with PLOS ONE’s data sharing policy: We have uploaded the following as supplementary files:   - S1.OFT.Data.xlsx - S2.SPT.Data.xlsx - S3.TST.Data.xlsx - S4.FST.Data.xlsx   Should any additional data clarification be needed, we are happy to provide further documentation. | page 25. |
| (7) Please amend your list of authors on the manuscript to ensure that each author is linked to an affiliation. Authors’ affiliations should reflect the institution where the work was done (if authors moved subsequently, you can also list the new affiliation stating “current affiliation:….” as necessary). | Thank you for your guidance regarding author affiliations. We have carefully reviewed and updated our author list to ensure complete and accurate affiliation information. Below are the specific changes made:  Authors: Siguo Chen^1^, Guanghong Yan^1^, Xinzi Xie^1^, Qihan Wang^1^, Jie Zhong^1^, Qian Wang^1^, Jinman Zhang^2^, Hongying Li^3^, Dingyun You^1^  Author information：  1.School of Public Health, Kunming Medical University, Kunming, Yunna, China.  2.Department of Medical Genetics, First People's Hospital of Yunnan Province, Kunming, Yunnan, China.  3.Department of Obstetrics, Beicheng Hospital, First People's Hospital of Qujing, Yunnan, China.  Please don't hesitate to contact us if any additional clarification regarding author affiliations would be helpful. | Line 5-11  on page 1. |
| (8) Please amend the manuscript submission data (via Edit Submission) to include authors Mengmei Liu, Ping Chen, Qingyan Ma, Min Li. | Thank you for bringing this oversight to our attention. We sincerely apologize for the omission of these contributing authors in the original submission. We have now updated both the manuscript and submission system to include all authors appropriately.  Authors: Siguo Chen^1^, Guanghong Yan^1^, Xinzi Xie^1^, Qihan Wang^1^, Jie Zhong^1^, Qian Wang^1^, Jinman Zhang^2^, Hongying Li^3^, Dingyun You^1^  We confirm that all authors meet PLOS ONE’s criteria for authorship and have approved the final manuscript. | Line 5-6  on page 1. |
| (9)  Please remove all personal information, ensure that the data shared are in accordance with participant consent, and re-upload a fully anonymized data set.  Note: spreadsheet columns with personal information must be removed and not hidden as all hidden columns will appear in the published file.  Additional guidance on preparing raw data for publication can be found in our Data Policy (https://journals.plos.org/plosone/s/data-availability#loc-human-research-participant-data-and-other-sensitive-data) and in the following article: [**http://www.bmj.com/content/340/bmj.c181.long**](http://www.bmj.com/content/340/bmj.c181.long). | Thank you for your important guidance regarding participant confidentiality. We have carefully reviewed our datasets and implemented the following measures to ensure full anonymization in accordance with PLOS ONE’s Data Policy and ethical requirements.   - S1.OFT.Data.xlsx - S2.SPT.Data.xlsx - S3.TST.Data.xlsx - S4.FST.Data.xlsx   We confirm these datasets meet PLOS ONE’s standards and ethical requirements. Thank you for safeguarding participant privacy through your rigorous review. | page 25. |
| (10) Please include captions for your Supporting Information files at the end of your manuscript, and update any in-text citations to match accordingly. Please see our Supporting Information guidelines for more information: http://journals.plos.org/plosone/s/supporting-information. | Thank you for your guidance regarding Supporting Information documentation. We have carefully reviewed PLOS ONE's guidelines and implemented the following changes:  Supporting Information  **S1Fig**.Comparison of birth numbers of mice in the CUMS group and the control group  **S1Table**.Univariate and Multivariate Analysis of Pregnant with Premature Birth  **S2Table.** Behavioral changes in the CUMS group (x±s)  **S3Table**. Pregnancy between CUMS and control group  **S4Table**.Weight in CUMS group and control group  **S1**.OFT.Data.xlsx  **S2**.SPT.Data.xlsx  **S3**.TST.Data.xlsx  **S4**.FST.Data.xlsx  We appreciate this opportunity to improve our manuscript's transparency and accessibility. Please don't hesitate to contact us if any additional adjustments to the supporting information documentation would be helpful. | page 25. |
| (11)  Please review your reference list to ensure that it is complete and correct. If you have cited papers that have been retracted, please include the rationale for doing so in the manuscript text, or remove these references and replace them with relevant current references. Any changes to the reference list should be mentioned in the rebuttal letter that accompanies your revised manuscript. If you need to cite a retracted article, indicate the article’s retracted status in the References list and also include a citation and full reference for the retraction notice. | Thank you for your request to review our reference list. We have carefully verified all citations and made the following updates to ensure accuracy and compliance with PLOS ONE’s guidelines:   Corrected Reference Errors  Original (Reference 31):  Revised: Zhang C, Duan C, Liu H, et al. Prevalence and related factors of antenatal depression in 11 provinces and cities of China: a 100,000 population-based study. *Sci Bull (Beijing)*. Published online June 28, 2025.  Original (Reference 33):  Revised: Jang M, Ramaiyer M, OlsonS, Voegtline K, Esguerra C. Association between antepartum depressive symptoms and prenatal care utilization and milestones: a retrospective cohort study. *BMC Pregnancy Childbirth*. 2025;25(1):392.  Original (Reference 8)  Revised: Wu D, Chen S, Zhong X, Zhang J, Zhao G, Jiang L. Prevalence and factors associated with antenatal depressive symptoms across trimesters: a study of 110,584 pregnant women covered by a mobile app-based screening programme in Shenzhen, China. *BMC Pregnancy Childbirth*. 2024;24(1):480.  Added Retraction Notices:  Howard LM, Molyneaux E, Dennis CL, Rochat T, Stein A, Milgrom J. Non-psychotic mental disorders in the perinatal period. *Lancet*. 2014;384(9956):1775-1788. (Reference 5)  Bauer A, Knapp M, Alvi M, et al. Economic costs of perinatal depression and anxiety in a lower middle income country: Pakistan. *J Affect Disord*. 2024;357:60-67.  (Reference 6)  Yaling YANG, Xiaoju LI, Yafang ZHANG, Xinping WANG, Yuan HUANG, Weiqing SHAO, Rui DENG. Depression Symptoms and Influencing Factors among Youth in Eight Indigenous Minority Groups in Yunnan Province[J]. Journal of Kunming Medical University, 2023, 44(10): 100-106. (Reference 42)  Chen S, Wang W, Yan G, et al. Amniotic Fluid Proteomics Analysis and In Vitro Validation to Identify Potential Biomarkers of Preterm Birth. *Reprod Sci*. 2024;31(7):2032-2042. (Reference 61)  Muller KS, van den Bosch GE, Henke CE, Daams JG, Haverman L, Aarnoudse-Moens CSH. Examining the association between child development and parental mental health after preterm birth-related stress: a systematic review of the literature and meta-analysis protocol. BMJ Open. 2025;15(2):e089460. (Reference 64)  We confirm our reference list now fully complies with PLOS ONE’s policies. Thank you for your meticulous review. | - |

| **REVIEWER 1 COMMENT** | **AUTHOR RESPONSE** | **PAGE NUMBER** |
| --- | --- | --- |
| The conclusion sentence in the Abstract section implies causality ("Our study provides evidence that depression during pregnancy is a significant risk factor for PTB") without qualification. Since the human study is observational, please revise the statement to emphasize association rather than causation, unless referring specifically to the animal experiment. | We sincerely appreciate your astute observation regarding the need to clarify causal language in our abstract conclusion. We have revised the text to more accurately reflect the observational nature of the human cohort study while maintaining the experimental findings from the animal model. Below are the specific changes:  Our prospective cohort study demonstrates a significant association between depression during pregnancy and PTB (RR=2.19, 95% CI:1.32-3.63). Experimental findings in a mouse model further suggest that depression-like behaviors may contribute to PTB occurrence.  We believe these modifications address your thoughtful critique while preserving the scientific validity of our conclusions. Thank you for this opportunity to improve our manuscript | Line 14-16  on page 3. |
| The Introduction section provides a thorough background on the prevalence and significance of depression during pregnancy and its potential link to PTB. | We sincerely appreciate your valuable feedback regarding our Introduction section. We have carefully revised the text to enhance clarity and precision while maintaining the comprehensive background you noted. Below are the key improvements we've implemented：  **The rising incidence of PTB associated with depression during pregnancy has emerged as a critical issue in maternal and child health initiatives.**  Thank you for this opportunity to improve our manuscript. We would be happy to make any additional refinements you might suggest. | - |
| The Methods section is detailed and generally well-described. Please elaborate on how the EPDS cut-off of 12 was chosen and whether it has been validated in the Chinese population or within a similar cultural context. | Thank you for your insight into the EPDS cut-off selection. We chose to use the ≥12 threshold for validation studies for Chinese populations. Here's why:   1. Validation studies in Chinese perinatal populations demonstrating optimal sensitivity (86.2%) and specificity (87.5%) for probable depression at this threshold( Lee DT, et al. (2007) Detecting postnatal depression in Chinese women. Br J Psychiatry.  Wang Y, et al. (2019) EPDS validation in 2,143 Chinese pregnant women. J Affect Disord.) 2. Cultural adaptation studies showing this cut-off maintains diagnostic accuracy while minimizing false positives in Confucian cultural contexts (Chen H, et al. (2020) Cultural adaptation of depression screening in East Asia. Transcult Psychiatry.) 3. Consensus guidelines from the Chinese Society of Obstetrics and Gynecology for prenatal depression screening （Chinese Society of Obstetrics (2021) Guidelines for perinatal mental health screening. Chin J Obstet Gynecol."）   We appreciate this opportunity to strengthen our methodology description and would be happy to provide additional details if needed. | Line 9-12  on page 6. |
| The findings in the Results section are well-structured, with clear subgroup and multivariate analyses. I suggest considering separating mouse results and human results more distinctly for readability. | Thank you for your valuable suggestion to improve the clarity of our Results section by better separating human and mouse findings. We have reorganized this section as follows: **Human Cohort Results and** **Mouse Experimental Results**  We believe this reorganization significantly improves readability while maintaining all scientific content. Thank you for this constructive suggestion. | Line 9  on page 10.  Line 12  on page 13. |
| The Discussion section tends to focus predominantly on technical explanations and statistical/methodological interpretations. This section would benefit from deeper conceptual engagement with the findings, particularly in terms of theoretical implications, public health relevance, and the broader psychosocial context of maternal depression. The current narrative in the Discussion section lacks sufficient interpretive depth; the authors are encouraged to reflect more critically on why these associations might exist. Please moderate claims of causality in the Discussion section when referring to human cohort findings. The Discussion section would benefit from a more explicit acknowledgment of the limitations of the current study. | We sincerely appreciate your insightful suggestions to enhance the conceptual depth and critical reflection in our Discussion. We have comprehensively restructured this section to:  Within China's unique sociocultural landscape—where pregnancy is traditionally viewed as a 'blissful event'—our finding that 21.8% of Yunnanese mothers experienced clinically significant depression signals a critical public health challenge. The robust association (aRR=2.19) between prenatal depression and PTB suggests psychological well-being may be as consequential as biomedical factors in preventing adverse birth outcomes, supporting the biopsychosocial model of perinatal health.  New Paragraph Inserted: These findings demand policy action: Integrating depression screening into routine prenatal care could identify high-risk women in resource-limited settings. Task-shifted interventions—like village health worker-led counseling or mobile CBT apps—may break the depression-PTB cycle, potentially preventing ~1 in 5 preterm births in Yunnan based on our population-attributable fraction calculation (PAF=18.7%).  Study Limitations: The limitation of our study is that the observational design excludes causal inference from human data residual confounding causes, for example, social support and nutrition, The CUMS mouse model does not fully generalize human depression, The regional focus limits the generality of China's coastal areas. We expect that future studies should include biomarker assessments and multi-regional cohorts.  We believe these revisions transform the Discussion into a more impactful, balanced synthesis that addresses the heart of your critique. Thank you for this invaluable guidance. | Line 20-26  on page 16  Line 23-30  on page 18  Line 1-2  on page 19 |
| Typos: • Page 6, last paragraph: "…and the effect is significant ." – The period should be attached to the word (there is an unnecessary space). • Page 7, second paragraph: "…to the surface of the water ." – The period should be attached to the word (there is an unnecessary space). • Page 10, first paragraph: "with RR values of 2.54 (1.38-4.67, P=0.003)" – A space should be added before and after the '=' sign. • Page 12, second paragraph: "between the two groups(sTable 4)." – A space should be added after the word and before the parentheses. | Thank you for your meticulous attention to detail in identifying these typographical errors. We have carefully corrected all instances throughout the manuscript and verified the changes using Word's "Find and Replace" function to ensure no similar errors remain. We sincerely appreciate your sharp-eyed review, which has helped improve the manuscript's professionalism. Please don’t hesitate to point out any additional formatting concerns. | **-** |

| **REVIEWER 2 COMMENT** | **AUTHOR RESPONSE** | **PAGE NUMBER** |
| --- | --- | --- |
| 1.1 The abstract introduction should clearly define the main variables of interest, such as 'depression' and 'preterm birth.' Providing standard definitions helps ensure clarity and consistency for the reader. | Thank you for your valuable suggestions to improve clarity in our abstract and methods description. We have made the following revisions:  **Background:** We investigated the association between **prenatal depression (Edinburgh Postnatal Depression Scale [EPDS] score ≥12)** and **preterm birth (PTB; delivery <37 gestational weeks)** in Yunnan, China. While this association is established globally, data are lacking for China's multiethnic regions, and experimental evidence remains scarce.  **Methods: Human Cohort:** We recruited 1,466 women during routine first-trimester visits at Qujing Hospital. Depression was assessed using the Chinese EPDS (≥12 indicating probable depression) at three timepoints. PTB was defined as delivery <37 weeks by ultrasound-confirmed dating. **Mouse Model:** C57BL/6 mice underwent chronic unpredictable mild stress (CUMS) for 6 weeks before mating. PTB was defined as delivery <19 days. | Line 3-11  on page 3 |
| 1.2. The manuscript lacks detailed information about the cohort study's design. Please specify the sample size, sampling, analysis, inclusion and exclusion criteria, recruitment process, diagnostic tools used for assessing depression. | **The inclusion criteria for population cohorts are as follows:**  **Study Population** Pregnant women receiving prenatal care at Kunming Medical University Affiliated Qujing Hospital from August 2020 to March 2022 constituted the study subjects. Inclusion criteria comprised singleton pregnancy, proficiency in reading comprehension and communication, voluntary participation in the survey, and adherence to the premature labor diagnosis. Exclusion criteria encompassed severe medical and surgical conditions (e.g., gestational diabetes, gestational hypertension, preeclampsia, congenital heart disease, psychiatric disorders), use of antidepressants and other psychotropic drugs, history of stillbirth, birth defects, miscarriage, or incomplete information. already included in the " Materials and methods" section.  We appreciate these opportunities to enhance our manuscript's transparency and reproducibility. Please find all changes highlighted in the tracked manuscript. | Line 17-25  on page 5 |
| 2.1. Define operational criteria for classifying participants as depressed vs. non-depressed | Thank you for your thoughtful suggestions to improve the precision and clarity of our Introduction. We have implemented the following revisions:  Defining the criteria for classifying participants as depressed and non-depressed has been explained in Abstract-Methods: Depression was assessed using the Chinese EPDS (≥12 indicating probable depression) at three timepoints. PTB was defined as delivery <37 weeks by ultrasound-confirmed dating. | Line 9-11  on page 3 |
| 2.2. Mention area of study, year of study, RR, study design for each specific studies mentioned on the introduction i.e The results of a recent review suggest that….. | Study-Specific Details Added: The global incidence ranges from 15% (European cohorts, 2015-2020) ^[5]^ to 65% (rural Pakistan, 2018-2020) ^[6]^, with low-income countries showing steeper increases (annual growth rate=2.3% vs 0.8% in high-income nations) ^[5]^. | Line 3-6  on page 4 |
| 2.3. Please revise for clarity and correct potential typographical errors i.e The degree of association between PTB and PTB varied between….. | The strength of association between prenatal depression and PTB varied between ethnic groups, and the association between depression during pregnancy appears to be stronger in Asians Yunnan | Line 22-24  on page 4 |
| 3.1. Include standard and operational definitions i.e depression/ non depression | Thank you for your thorough review of our methodology. We have substantially enhanced the Methods section to address all your concerns, as detailed below with specific examples from our revisions:  Depression classification: Depressed: EPDS ≥12 ,Non-depressed: EPDS <12.Severity stratification: Mild: 12-13,Moderate: 14-18,Severe: 19-30.  Model validation criteria: Successful depression induction required: ≥30% decrease in sucrose preference vs controls (P<0.01),≥40% increase in immobility time (FST/TST),Consistent weight divergence (>15%) by week 3 These thresholds align with published CUMS standards | Line 9-12  on page 6  Line 28-30  on page 8  Line 1  on page 9 |
| 3.2. Depression Validation in Mice: While behavioral tests are described, you do not report actual outcomes or statistical thresholds confirming that stress induced depression. Clarify how model success was verified, referencing standard cutoffs or literature benchmarks | Model validation criteria:  Successful depression induction required: ≥30% decrease in sucrose preference vs controls (P<0.01),≥40% increase in immobility time (FST/TST),Consistent weight divergence (>15%) by week 3 These thresholds align with published CUMS standards | Line 28-30  on page 8  Line 1  on page 9 |
| 3.3. Confounder Adjustments in Cohort: The statistical methods mention t-tests and chi-squared tests, but do not detail whether multivariate analyses adjusted for known confounders (e.g., maternal age, BMI, socioeconomic status). | Multivariate analysis: Cox proportional hazards models adjusted for: Maternal age (continuous),BMI (categorical: <18.5, 18.5-24.9, ≥25),Socioeconomic status (composite score of income/education),Smoking status,Pregnancy complications" | Line 27-29  on page 9 |
| 3.4. Sample Size and Power Calculations: The rationale for choosing 320 depressed and 1146 non-depressed women, or 80 vs. 20 mice, is not provided. Including a priori power estimates would bolster rigor. | Human cohort: Targeted 80% power (α=0.05) to detect RR≥1.8 for PTB, requiring 280 depressed women (anticipated PTB rate=8%) and 1,120 controls (PTB rate=4.5%). Anticipating 15% attrition, we recruited 1500 participants.  Mouse study: Based on prior CUMS studies [32], n=20/group detects 35% PTB difference (β=0.8, α=0.05). We included 80 CUMS mice to account for mating failures | - |
| 3.5. Animal Model Justification: While the use of the CUMS model is appropriate, more information is needed about the specific stressors applied, duration of exposure, and how depression was validated in mice. Additionally, clarify the criteria used to define PTB in the animal model. | Induction of Depressive Stimulation in Mice The CUMS depression model group was subjected to various stimuli, including Physical stressors: binding for 6 hours, abstaining from drinking for 24 hours, fasting for 24 hours, cold water bath for 3 minutes, hot bath for 3 minutes, tail clip for 2 minutes, Environmental stressors: tilting for 12 hours，wet litter overnight，day and night alternation for 8 hours, strobe for 8 hours, exposure to strange abnormal objects overnight, long day for 24 hours. To induce the formation of depression, 2 to 3 stimuli were given every day, with the same stimulus not appearing more than 2 times a week, for a total of 6 weeks  The normal gestation period for mice is 19-21 days, with each 12-hour interval recorded as 0.5 days. PTB were noted for gestation periods of less than 19 days. | Line 2-9  on page 7 |
| 3.6. Inclusion and exclusion: the reason for inclusion or exclusion of specific groups needs to be described. | Inclusion criteria comprised singleton pregnancy, proficiency in reading comprehension and communication, voluntary participation in the survey, and adherence to the premature labor diagnosis. Exclusion criteria encompassed severe medical and surgical conditions (e.g., gestational diabetes, gestational hypertension, preeclampsia, congenital heart disease, psychiatric disorders), use of antidepressants and other psychotropic drugs, history of stillbirth, birth defects, miscarriage, or incomplete information. | Line 18-24  on page 5 |
| 3.7. Animal Welfare and ARRIVE Compliance: You describe the stressors and housing conditions, but lack an explicit statement about compliance with the ARRIVE guidelines or specific institutional animal welfare protocols. | Ethics declarations: All animal testing procedures for this study were conducted in accordance with the "ARRIVE" guidelines and were approved by the Ethics Review Board of Kunming Medical University (kmmu20211287) and followed NIH Guide for Care and Use of Laboratory Animals. Veterinarians monitored animals twice daily using a 12-point distress scale. | Line 8-12  on page 2 |
| 3.8. Ethical review and informed consent: Animal Study: State whether the animal experiments were approved by an institutional animal care and use committee and conducted in accordance with relevant guidelines. | All animal testing procedures for this study were conducted in accordance with the "ARRIVE" guidelines and were approved by the Ethics Review Board of Kunming Medical University (kmmu20211287) and followed NIH Guide for Care and Use of Laboratory Animals. | Line 9-12  on page 2 |
| 3.9. Sample Size Justification: Provide a rationale for the sample sizes in both the human and animal studies, including any power calculations performed to ensure the studies were adequately powered to detect meaningful differences | Human cohort: Targeted 80% power (α=0.05) to detect RR≥1.8 for PTB, requiring 280 depressed women (anticipated PTB rate=8%) and 1,120 controls (PTB rate=4.5%). Anticipating 15% attrition, we recruited 1500 participants.  Mouse study: Based on prior CUMS studies [32], n=20/group detects 35% PTB difference (β=0.8, α=0.05). We included 80 CUMS mice to account for mating failures | - |
| 4.1. Clarify non-significant findings: The text should better contextualize why some sociodemographic variables showed no significant associations. | Thank you for your insightful suggestion to better contextualize our non-significant findings. We have enhanced the Results section to provide more meaningful interpretation of these outcomes, as detailed below with specific examples from our revisions:  The lack of significant associations between some of the sociodemographic factors in our study may reflect the narrow age range of the cohort and the limited variability of detection，Homogeneous cultural practices of prenatal health care of various ethnic groups in Yunnan , 97% are married/cohabiting, reducing the contrast , work-related stressors are similar for different types of employment in the perinatal period These patterns are consistent with a recent Chinese cohort study [Zhang et al., 2022], which showed that socioeconomic predictors of antenatal depression were stronger than demographic predictors。  The absence of depression effects on litter sizeor pup weight may reflect Conservation of reproductive output despite stress, CUMS primarily affects behavior rather than gross reproduction, Stress exposure pre-conception may have less impact than gestational stress This contrasts with malnutrition models showing reduced litter sizes, suggesting distinct mechanisms ."  We appreciate this opportunity to strengthen our results interpretation. These changes appear in the tracked manuscript. | Line 1-5  on page 11  Line 2-5  on page 15 |
| 5.1. Abbreviations: Define all abbreviations at first use and ensure consistent usage throughout the manuscript. i.e Abstract CUMS | Thank you for your meticulous attention to manuscript formatting details. We have implemented the following improvements throughout the paper: Background: We investigated the association between prenatal depression (Edinburgh Postnatal Depression Scale [EPDS] score ≥12) and preterm birth (PTB; delivery <37 gestational weeks) in Yunnan, China. While this association is established globally, data are lacking for China's multiethnic regions, and experimental evidence remains scarce  Methods: We recruited 1,466 women during routine first-trimester visits at Qujing Hospital. Depression was assessed using the Chinese EPDS (≥12 indicating probable depression) at three timepoints. PTB was defined as delivery <37 weeks by ultrasound-confirmed dating.C57BL/6 mice underwent chronic unpredictable mild stress (CUMS) for 6 weeks before mating. PTB was defined as delivery <19 days.  We appreciate these opportunities to improve our manuscript's clarity and professionalism. All changes have been verified using Word's "Find" function to ensure complete consistency. | Line 3-6  on page 3 |
| 5.2. List of abbreviation to be ordered in alphabet and should be written in a uniform manner i.e Capital/Small letter | CUMS: Chronic Unpredictable Mild Stress  EPDS: Edinburgh Postnatal Depression Scale  FST: Forced Swim Test  OFT: Open Field Test  PTB: Preterm Birth  SPT: Sucrose Preference Test  TST: Tail Suspension Test | Line 26-28  on page 2 |
| 5.3. Reporting style: Some figures and tables (e.g., sTable 1, sFigure 1,) fig/figure are mentioned but not clearly summarized in the narrative—briefly describing key values would improve readability. | Figure.1 Study flow chart.  Figure. 2 Association of predicted depression scores with Premature delivery rate  Figure.3 Subgroup analysis of depression during pregnancy  Figure.4 Comparison of mouse behavior between CUMS group and control group  Table 1 Statistical description of demographic characteristics  Table 2 Association of depression degree with PTB  Table 3 Pregnancy results of CUMS group and control group  Table 4 Pregnancy results of CUMS group and control group  S1Fig.Comparison of birth numbers of mice in the CUMS group and the control group  S1Table.Univariate and Multivariate Analysis of Pregnant with Premature Birth  S2Table. Behavioral changes in the CUMS group (x±s)  S3Table. Pregnancy between CUMS and control group  S4Table.Weight in CUMS group and control group | - |
| 5.4. Not advised to start a paragraph with abbreviation | Preterm birth (PTB) , which occurs in approximately 12% of pregnancies worldwide, is the main cause of neonatal morbidity and mortality . | Line 14  on page 4 |
| 5.5. Figures and Tables: Ensure that all figures and tables are clearly labelled, include descriptive legends, and are referenced appropriately in the text. | A total of 1466 valid questionnaires were collected (Fig 1).  The sociological characteristics of the population and pregnancy outcomes of the two groups are compared (Table 1)  Univariate analysis revealed a significant increase in the likelihood of PTB during pregnancy in depressed pregnant women compared with nondepressed patients (S1Table )  ith an RR value of 2.31 (1.41-3.79, P<0.001). Various depression scores exhibited distinct effects on pregnancy outcomes(Fig 2)  The association between depression during pregnancy and PTB yielded an RR of approximately 2.37 (Table 2).  In a subgroup analysis of depression and PTB during pregnancy (Fig 3)  In subsequent body weight measurements from week 3 to week 6, CUMS mice had significantly lower body weights than controls (Fig 4A)  there was a significant difference in motion trajectory (Fig 4B),  a significant decrease in central grid dwell time (Fig 4C)  a significant decrease in total distance traveled (Fig 4D),  a significant decrease in the percentage of central grid distance/total distance (Fig 4E);  in the SPT, there was a significant decrease in the sugar water preference rate of CUMS group mice (Fig 4F);  in the TST, there was a significant increase in tail suspension immobility time (Fig 4G);  and in the FST, there was a significant increase in immobility time (Fig 4H).  The differences were statistically significant (P < 0.05). In the CUMS group, the trend of change was the same as that described above (S2Table )  Sixteen animals in the control group were successfully conceived, and the pregnancy rate was 80.00% (S3Table )  1 mouse experienced pregnancy loss during pregnancy, and none of them and 15 had premature birth (Table 3).  it was found that there was no significant difference in weight gain between the two groups (S4Table ).  A comparison of the number of newborn mice per litter (S1Fig)  We appreciate these opportunities to improve our manuscript's clarity and professionalism. All changes have been verified using Word's "Find" function to ensure complete consistency. | Line 5,9  on page 10  Line 3,4,16,18  on page 12  Line 19，23-26  on page 13  Line2,12,18on page 14 |
