# Supplementary material for: Association between depression during pregnancy and preterm birth: Results from population cohorts and mouse experimental models
Source: PLoS One. 2026 Jan 29;21(1):e0341449. doi: 10.1371/journal.pone.0341449 (PMC12854446; doi:10.1371/journal.pone.0341449)
Supplement: Attachment — Submitted filename: Response to Reviewers.doc [file pone.0341449.s012.doc]

# Author response to academic editor and reviewers

MANUSCRIPT ID: PONE-D-24-60622

MANUSCRIPT TITLE: Association between depression during pregnancy and preterm birth: results from

population cohorts and mouse experimental models

| **academic editor** | **AUTHOR RESPONSE** | **PAGE NUMBER** |
| --- | --- | --- |
| Major concern: Why was pre-pregnancy BMI and parity not controlled for The study was conducted during COVID 19 global pandemic, so how well authors can say its because of antenatal dep, not bec of COVID 19 anxiety and depression. | **Why was pre-pregnancy BMI and parity not controlled for?**  We sincerely thank the reviewer for this critical suggestion. In the original submission, data on pre-pregnancy BMI and parity were not available for the entire cohort. However, in direct response to this comment, we undertook a thorough review of medical records and successfully retrieved pre-pregnancy BMI data and parity data of our participants.  Following the reviewer's recommendation, we have now expanded our statistical analysis to include three models:  **Model 1:** no adjustment for confounding factors .  **Model 2:** adjust age, nationality, residence, marital status, education level, occupation status, annual family income, pregnancy.  **Model 3 :** adjust for all above + pre-pregnancy BMI and parity  We are pleased to report that the association between prenatal depressive symptoms and preterm birth remained statistically significant and robust across all models. Specifically, in the fully adjusted Model 3, the adjusted Risk Ratio was 2.08 (1.25,3.47). This result strengthens our primary conclusion that the association is independent of these important biological and obstetric factors.The results of all three models are now presented in the revised **Table 2** of the manuscript. We have also updated the Statistical Analysis section in the Methods to describe this new modeling strategy. We believe this comprehensive analysis directly addresses the reviewer's concern and significantly enhances the strength of our findings.  **The study was conducted during COVID-19 global pandemic, so how well authors can say its because of antenatal depression, not because of COVID-19 anxiety and depression?**  This is a very insightful comment. We agree that the COVID-19 pandemic constituted a pervasive population-level stressor that likely elevated the baseline levels of anxiety and depressive symptoms in our cohort, as it did globally.  However, we believe our findings regarding the association between prenatal depression and PTB remain valid for the following reasons:  Focus on the Mediating Pathway: Our study examines the association between the psychological state (depression, measured by EPDS) and the pregnancy outcome (PTB). The COVID-19 pandemic can be viewed as a distal trigger that contributed to the population's risk of developing this psychological state. The core of our hypothesis is that once a depressive state is established, it exerts a biological effect (e.g., HPA axis dysregulation) that increases PTB risk, irrespective of the initial trigger.  Internal Validity: Crucially, the external stressor of the pandemic was a background condition affecting all participants equally. The comparison in our study is within the cohort during the same period: women with high EPDS scores (≥12) versus women with low EPDS scores (<12). The significantly higher incidence of PTB in the high EPDS group suggests that the individual's psychological response to the overarching stressor (i.e., developing clinically significant depression), rather than the stressor itself, is the key factor associated with the adverse outcome. This design preserves the internal validity of our observed association.  Supporting Literature: Emerging research conducted during the pandemic has similarly reported independent associations between prenatal mental distress and adverse birth outcomes, reinforcing the idea that the mental health state itself is a critical risk factor (e.g.,  [PMID: 36533427] & [PMID: 38712806]).  We have revised the discussion to directly address this point and acknowledge the context of the pandemic . | Page 14  Line 10-25 on page 18. |
| Abstract: Can you briefly add a background and then redirect to your study aims. Add risk of preterm birth Remove the bold from aims. Add in methods of abstract, add depression symptoms, as EPDS is not a diagnostic tool rather it’s a screening tool. Rewrite the results in the abstract properly and define RR. Do refer how others write results. Don’t add RR and values in conclusion Revise the conclusion currently you’re just writing the results again. Add few lines of overall results and provide a suggestion and point to the future direction. | We sincerely thank the editor and reviewers for their time and valuable comments, which have greatly helped us to improve the quality of our manuscript. We have made the following revisions to the abstract section.  **Background:** Depression is a prevalent psychological challenge during pregnancy, with established links to adverse outcomes like preterm birth (PTB) globally. However, epidemiological data from China's multiethnic regions are scarce, and experimental evidence supporting a causal relationship remains limited. This study aimed to investigate the association between prenatal depressive symptoms and the risk of PTB in a cohort from Yunnan, China, and to provide supportive evidence using a mouse model of depression.  **Methods:** We recruited 1,466 women during their first-trimester routine visits at Qujing Hospital. Depressive symptoms were assessed using the Chinese version of the Edinburgh Postnatal Depression Scale (EPDS), a screening tool, with a score ≥12 indicating elevated symptoms suggestive of depression. PTB was defined as delivery before 37 gestational weeks, confirmed by ultrasound. In parallel, a mouse model of depression was established using Chronic Unpredictable Mild Stress (CUMS) for 6 weeks prior to mating. PTB in mice was defined as delivery before 19 days of gestation.  **Results:**In the cohort study, the incidence of PTB was significantly higher in women with prenatal depressive symptoms compared to those without (8.43% vs. 3.83%, P < 0.001). The association remained significant after adjusting for sociodemographic and clinical confounders, with an adjusted risk ratio (aRR) of 2.19 (95% CI: 1.32-3.63). This association showed a significant dose-response pattern (P for trend = 0.03), with the risk being highest for women with moderate depressive symptoms (aRR = 2.44, 95% CI: 1.30-4.58). In the animal experiments, PTB did not occur in the control mice, whereas 40% of the mice exposed to CUMS (depression model group) delivered prematurely.  **Conclusion:** This study demonstrates a significant association between prenatal depressive symptoms and an increased risk of preterm birth in a Chinese multiethnic cohort. Experimental findings from a mouse model further suggest a potential contributory role of depression to PTB. These results underscore the importance of screening for and addressing maternal mental health during pregnancy. Future research should focus on underlying mechanisms and intervention strategies to mitigate this risk. | Line 25-29 on page 3 and Line 1-26 on page 4. |
| General comments: Don’t state literature…in your text. Use previous studies Introduction What is meant by today? In the introduction add 2025 if it’s so. Along with months. I quickly checked the reference, but the reference is until 2021? In the second para, spell what are the multiple risk factor? In the same para you have described how it’s going to affect, add 1-2 lines as it’s the major objective of the study. I am not clear by these statements what authors want to say. “The strength of association between prenatal depression and PTB varied between ethnic groups, and the association between depression during pregnancy appears to be stronger in Asians Yunnan ^[25-27]^, as a multiethnic region, has not reported the association between depression during pregnancy and PTB, and it remains to be determined whether the effect of depression on PTB is different from that found in other regions”  Add rationale why do you think it varies with different ethnic origin. The major missing is what is the rationale behind the use of animal models, add a paragraph explaining previous studies on animals’ models, what they found with respect to PTB and also report studies looking at antenatal depression on fetal brain development.  Add details of what is need for looking at a population from Yunnan. How does it vary from other Chinese province.  If you had any prior hypothesis add that too. | We agree with the reviewer's suggestion for more precise language. We have replaced the term "literature" with "previous studies" or similar phrases throughout the manuscript to improve clarity and professionalism.  We thank the reviewer for pointing out this imprecision. We have removed the vague term "today" and rephrased the sentence to more accurately reflect the timeline of the existing evidence, as suggested.  This is a valuable suggestion to strengthen the logical flow of our introduction. We have now specified examples of risk factors and, as recommended, added a sentence to more explicitly connect the established knowledge about depression as a risk factor to the primary objective of our present study.  We appreciate the reviewer's feedback regarding the lack of clarity in this paragraph. We have thoroughly rewritten the problematic sentence to separate the distinct points and improve logical sequencing. Additionally, we have incorporated a rationale for why the association might vary by ethnicity, as requested, by mentioning potential contributing factors such as sociocultural and genetic influences. This revision, we believe, provides a much clearer and more compelling justification for conducting our study in Yunnan's multiethnic population.  We believe these additions have substantially enhanced the clarity, depth, and scholarly rigor of our introduction. | Line 11on page 5 and Line 21-36 on page 5  Line 7-19  on page 6. |
| Methods: I think you need to keep it as women with depressive symptoms, don’t just keep it as depressive alone it confuses. Given the lack of my expertise in animal models I am not comments on animal model part, I have seen reviewers with that expertise have commented. | We thank the reviewer for this critical comment. We agree that precise terminology is paramount. Throughout the manuscript, particularly in the Methods and Results sections, we have replaced potentially misleading terms such as "depressive" and "non-depressed" with more accurate descriptions based on the screening instrument used. The groups are now consistently referred to as "women with elevated depressive symptoms (EPDS ≥ 12)" and "women without elevated depressive symptoms (EPDS < 12)". We have also added explicit statements in the 'Screening Tools' subsection clarifying that the EPDS is a screening tool and not a diagnostic instrument. We believe these changes significantly improve the clarity and accuracy of our work.  We thank the reviewer for their time and acknowledge their expertise lies elsewhere. As the reviewer noted, other reviewers with specific expertise in animal models have provided their comments separately, and we have addressed those points in detail in other parts of our response letter. | Line 1-2  on page 8. |
| Statistical analysis:  Can you add the assumption of Cox Proportion model. Why was BMI, parity, marital status not considered? I would strongly suggest adding those in the model. For e.g., marital status I can see it in the Table 1. But still it play a major role. I would suggest three models. 1. Crude model 2. Current model 3. Current model + BMI + Parity  Importantly, I am concerned that the recruitment happened during COVID 19 pandemic, how authors adjust control for it. See papers how they accounted for. <https://www.nature.com/articles/s41390-024-03620-7> <https://www.sciencedirect.com/science/article/pii/S2949732924000735>   Further in the screening tools for depression during pregnancy – when was the questionnaire administered. I can’t find any details of it. How many responded and did not. Add those in Fig 1. Revise as depressive symptoms don’t keep it as diagnosis | Response to Comments on Statistical Analysis and Methods:  Cox Model Assumption: We thank the reviewer for this suggestion. We have now explicitly stated that the proportional hazards assumption was tested using Schoenfeld residuals and was met, and we have included this in the revised Statistical Analysis section.  Model Specification and Additional Covariates: We agree with the reviewer that including pre-pregnancy BMI and parity strengthens the analysis. While these data were not initially available for all participants, we have since retrieved them from a subset of medical records for 89% of the cohort (n=1304). As recommended, we now present three models:  **Model 1:** no adjustment for confounding factors .  **Model 2:** adjust age, nationality, residence, marital status, education level, occupation status, annual family income, pregnancy.  **Model 3 :** adjust for all above + pre-pregnancy BMI and parity  The results, which remain robust across all models, are presented in the revised manuscript (Table 2, Page 14).  We sincerely thank the reviewer for raising this important point regarding the potential confounding effect of the COVID-19 pandemic. We fully acknowledge that the pandemic constituted a significant population-level stressor. We have carefully considered this issue and believe that our study design and complementary experimental evidence effectively address this concern, as detailed below.  1. Internal Comparison Within a Shared Context: The core of our analytical approach is an internal comparison within a cohort that shared the same macro-environmental context of the pandemic. The pervasive stress of COVID-19, as the reviewer rightly notes, elevated the background level of anxiety and depressive symptoms for all participants in our study. However, our analysis does not compare a "pandemic group" to a "non-pandemic group"; rather, it compares women with high depressive symptoms (EPDS ≥12) to women with low depressive symptoms (EPDS <12) within the same pandemic context.  If the pandemic environment alone were the primary driver of preterm birth (PTB), we would expect to see little to no difference in PTB rates between these two groups, as both were exposed to the same overarching stressor. The fact that we observed a robust and significant increase in PTB risk specifically in the group with high depressive symptoms strongly suggests that the individual's psychological vulnerability and response—namely, the development of significant depressive symptoms—is the critical mediator of PTB risk, over and above the general background stress.  2. Corroborating Evidence from Animal Experiments: Furthermore, our study is uniquely positioned to address this concern due to the inclusion of an animal model. The animal experiment was conducted in a controlled laboratory environment, entirely isolated from the human COVID-19 pandemic. In this setting, we induced depression-like behaviors in mice using the Chronic Unpredictable Mild Stress (CUMS) paradigm and observed a significantly higher rate of PTB compared to unstressed controls.  This finding is crucial because it demonstrates that a depression-like state can directly contribute to PTB in the absence of any pandemic-related factors. This experimental result provides independent, supportive evidence for a potential causal pathway from prenatal depression to PTB, which strengthens the interpretation of our human cohort findings.  3. Manuscript Revisions: In direct response to this comment, we have revised the Discussion section of our manuscript to explicitly acknowledge the pandemic context and articulate the above arguments. We have added a new paragraph that clarifies the logic of our internal comparison and integrates the supporting evidence from our animal model .We believe that this combination of a robust study design and compelling complementary data provides a strong foundation for our conclusion that prenatal depressive symptoms are associated with an increased risk of PTB, independent of the shared context of the COVID-19 pandemic.  Fig 1 We have completed the revisions as requested. | Page 14.  Line 10-25on page 18 |
| Results: “Notably, the incidence of depression in rural pregnant women was slightly higher than that in urban pregnant women, with depression incidence rates of 26.01% and 18.77%, respectively” Don’t compare it in results, just talk about your results, rest all move to discussion.  Same way remove this too “which may reflect the narrow age range of the cohort and the limited variability of testing, as well as the homogeneous cultural practices of prenatal care among ethnic groups in Yunnan”  In the results section just describe your results. Add crude and Model 2 results in your revision.  I can’t find what is the rationale for conduction degree of depressive symptoms. If you want to have those results, add it as aim in the introduction and add it as statistics too. I can see any bivariate analysis in the statistics…..Pls add. Also do multiple comparison corrections given the number of tests.,  I am unclear why authors did this tests (Subgroup analysis of depression and PTB during pregnancy) from where they have got the information for section trimester… Don’t add any new tests – if its post hoc specify it as posthoc. And I can’t find details of when was demographics questionnaire administered. Plus what is meant by “those in the second trimester (RR, 2.60; 95% CI, 1.18-5.70), with…  Overall, the results section needs clarity and rewrite the whole results sections. All the tests need to specify earlier in statistics and should be in the same order. Don’t do any fishing of tests.  I can’t find discussion subheading? Pls include.  What do you mean by preventive role “To our knowledge, this is the first report to explore the preventive role of depression during pregnancy on PTB in both human and animal studies”  In the discussion authors state that “Characteristics of the impact of depression on PTB in Yunnan. The detection rate of depression during pregnancy is higher than that reported in previous studies” Can this be due to COVID?  I can’t find detailed discussion about PTB among mothers with depression.  Add strengths | **Regarding the Results section:** “We have rewritten the entire 'Results' section to be purely descriptive. All interpretive statements, such as those comparing urban/rural incidence and speculating on non-significant findings, have been moved to the 'Discussion' section.”  **Regarding statistical methods:** “We have expanded the 'Statistical Analysis' subsection to detail all pre-planned analyses, including the three Cox models, the dose-response analysis, and the pre-specified subgroup analyses. We have also stated that a Bonferroni correction was applied for multiple comparisons in subgroup analyses.”  **Regarding subgroup analysis and pregnancy:** “We have clarified that the subgroup analysis was pre-planned and that the 'trimester' information came from the gestational age recorded at the first-trimester visit when the baseline demographic questionnaire and EPDS were administered.”  **Regarding the discussion section:** “We have structured the 'Discussion' with clear subheadings ('Principal Findings', 'Comparison with Previous Studies', etc.) and removed the incorrect term 'preventive role'. We have also added a dedicated 'Strengths and Limitations' paragraph.” | Page14、15、16 |
| **REVIEWER 3 COMMENT** | **AUTHOR RESPONSE** | **PAGE NUMBER** |
| Author contribution section Comment 1: In the author contribution section, you have described that authors have different contributions falling in two categories and it is better to describe the contribution of each author in words rather than simply describing as have equal contribution. Comment 2: Better to use a special character in the author list to indicate the corresponding author and no need of repeating the affiliation describing in author information to indicate corresponding author. Comment 3: Please remove or provide a reason for using “hyphen” for words in funding section. Comment 4: Please include full terms for the acronyms/abbreviation such as “ARRIVE’ and “NIH” (ethical consideration section) in your abbreviation section. | **Thank you for your careful attention to our format. As you mentioned, we have made the following adjustments:**  **Author Contributions:** **Siguo Chen:** Conceptualization, Methodology, Formal Analysis, Writing - Original Draft. **Guanghong Yan:** Investigation, Data Curation, Writing - Review & Editing. **Xinzi Xie:** Investigation, Resources, Validation. **Qihan Wang:** Software, Formal Analysis, Visualization. **Jie Zhong:** Project Administration, Investigation. **Qian Wang:** Resources, Data Curation. **Jinman Zhang:** Supervision, Methodology. **Hongying Li:** Resources, Supervision. **Dingyun You:** Supervision, Funding Acquisition, Writing - Review & Editing, Project Administration.  **Corresponding author**  Dingyun You  **Abbreviations**  **ARRIVE:** Animal Research: Reporting of In Vivo Experiments  **NIH:** National Institutes of Health  **SPF:** Specific Pathogen Free | Page1、3 |
| Abstract section Background section: No need of writing sentences in bold and use period(.)after the word scarce. Method section: Please provide information about sample size, sampling procedure, study design and sampling procedure and how you reach your samples. It is not recommended to write about the definition of what PTB mean in this section. There is also spacing error (line 4). Please revise it and made necessary correction. Introduction section The introduction section needs minor revision regarding word utilization such as (for example better to use “Low- and middle-income countries” instead of low-income and middle-income countries), and it also for spacing and punctuation marks utilization. | In the previous review, we have systematically rewritten the summary section.  In the previous review, we have systematically rewritten the abstract section and made the following modifications to the introduction: **Low- and middle-income countries**，we also checked the punctuation throughout the entire text. | Line 8  on page 4. |
| Material and Methods section Comment 1: Better to write the Ethical review and informed consent part of your manuscript at the end of methods and material section. Comment 2: As the methods section is the core of the study, your research project lacks clarity. In the methods section, the study design, sampling, sampling procedure, Data quality management, Source and study Population, operational definitions should be described in clear and concise way to make clear for the reader. Your research project needs revision regarding these issues. You have to write the details how you recruited 1466 participants from 1500 and your study flow chart (figure 1) is not also matched with this sample size. Please check it for more clarity. Comment 3: Please try to separate sub-headings from the rest of the paragraph following it. It is recommended to use either separate line or using colon. Comment 4: For what SPF is standing for? Please include it in your abbreviation section. Comment 5: Better to avoid words like PTB during pregnancy, simply if you mention PTB, it is known that it happens during pregnancy. So, no need of writing like “PTB during pregnancy”. Comment 6: Please provide the operational definitions for term Chronic Unpredictable Mild Stress (CUMS). | **We have made the following additions in the methodology section: Study Design and Population** This study combined a prospective cohort design with an experimental animal model**.**  **Data Quality Management:** To ensure data quality, all questionnaires were administered by trained staff. Electronic data entry was performed with built-in validation checks, and a random sample of 10% of the paper records was double-checked for accuracy.  **And adjustments were made to errors in format and terminology.** | Line29-32 on page 6.  Line11-15 on page 7. |
| Result and Discussion section Comment 1: Please describe in what unit the household monthly income is measured. Is it in $ US or other? Usually, it is recommended to describe it in US dollars. Comment 2: Please check the font size for headings and-sub headings. Usually, the font size for headings is larger than or equal with subheadings please check for that. Comment 4: please Insert Tables using caption and check spacing and punctuation marks as well. Comment 5: Is depression a risk factor or preventive factor for PTB? Please provide details about this sentence in your result “To our knowledge, this is the first report to explore the preventive role of depression during pregnancy on PTB in both human and animal studies. “Contradicts with conclusion of your study otherwise please briefly explain it. Comment 6: Your discussion part includes unnecessary details, please focus to elaborate more issues related to your study result. Comment 7: What are the Strengths and limitation of your study? | The modifications we made in the Results and Discussion section include: converting ¥120,000 to $16,500, adjusting the formatting of table headings, adding 'Strengths and Limitations' in the Discussion section, and systematically rewriting the Discussion section. | page 13、18、19. |
| **REVIEWER 4 COMMENT** | **AUTHOR RESPONSE** | **PAGE NUMBER** |
| 1. Scope and Inclusion Criteria • While the focus on quantitative studies is clear, excluding qualitative and mixed-methods research may limit the richness of the evidence base. Qualitative studies often provide valuable insights into paternal mental health experiences that may not be captured through quantitative measures. At minimum, the rationale for excluding qualitative studies should be clearly articulated. • Excluding studies where paternal outcomes are embedded within broader "parental" research may unintentionally omit valuable data. Consider allowing inclusion of such studies if fathers’ data are separately reported, even if paternal mental health is not the main focus. | We sincerely thank the reviewer for their positive and insightful comments on our manuscript. We have carefully considered each point and have revised the manuscript accordingly to strengthen our work. Our detailed responses are below. **Scope and Inclusion Criteria**  We thank the reviewer for this thoughtful suggestion. Our study was specifically designed as a quantitative investigation to measure the *association strength* and *potential causal relationship* between prenatal depression and preterm birth using statistical and experimental methods. While we fully agree that qualitative research provides invaluable insights into the lived experiences and perceptions of mothers, its methodological framework is distinct from our aim to generate generalizable, quantifiable effect estimates.  Therefore, to maintain methodological coherence and the specific objectives of our study, we focused exclusively on quantitative data from our cohort and experimental model. We have now clarified this rationale in the revised 'Methods' sectionas follows:  **This study used a combination of prospective cohort design and experimental animal models, and a quantitative study design that did not include qualitative methods because the primary objective was to quantify the association between depressive symptoms and risk of preterm birth, rather than to explore subjective experience.** | Page 6 |
| 2.Methodological Details • The planned use of JBI critical appraisal tools is appropriate. However, a brief justification for selecting JBI over other tools (e.g., CASP for qualitative studies, if those were to be included) would increase transparency. • The criteria for conducting a meta-analysis (based on I² thresholds) are clearly stated. However, the protocol would benefit from outlining potential subgroup analyses (e.g., by region, type of mental health outcome, or study design) to allow for a more nuanced synthesis if sufficient data are available. • Given the psychological nature of the outcomes, more explanation of why qualitative evidence is excluded would help readers understand the methodological boundaries of this review. • There are minor typographical and formatting inconsistencies (e.g., spacing, repeated phrases), which should be corrected for clarity. | **Methodological Details**  We appreciate the reviewer's suggestions to enhance our methodological transparency.  **Subgroup Analyses:** As recommended, we have now pre-specified our plan for subgroup analyses in the 'Statistical Analysis' section to explore potential effect modification. The revised text states:  **"If sufficient data are available from the cohort, pre-specified subgroup analyses will be conducted to examine the association between depressive symptoms and PTB across strata defined by maternal age (<25, 25-34, ≥35 years), socioeconomic status (low/high), and place of residence (urban/rural)."**  **Typographical Errors:** We have thoroughly proofread the manuscript and corrected minor typographical and formatting inconsistencies for improved clarity. | Page 12 |
| 3.Discussion Depth The revised discussion now includes sociocultural context, public health implications, and policy relevance. The addition of population-attributable fraction (PAF=18.7%) is particularly impactful. Further elaboration on potential mechanisms (e.g., HPA axis dysregulation, inflammation) could enrich the theoretical framing. | **Discussion Depth**  We thank the reviewer for this excellent suggestion. We have significantly enriched the 'Discussion' section by adding a new paragraph dedicated to potential biological mechanisms, which strengthens the theoretical framing of our findings.  **New text added to the 'Discussion' section line:**  **"Potential Mechanisms Linking Prenatal Depression and Preterm Birth"**  The biological mechanisms underlying the association between prenatal depression and PTB are likely multifactorial, involving complex neuroendocrine and immune pathways. One prominent pathway is the dysregulation of the maternal hypothalamic-pituitary-adrenal (HPA) axis. Chronic psychological stress and depression can lead to hypercortisolemia, and elevated cortisol may cross the placenta, potentially disrupting fetal development and triggering pro-inflammatory cascades that promote uterine contractility and cervical remodeling . Concurrently, depression is associated with a heightened state of systemic inflammation, characterized by increased levels of pro-inflammatory cytokines such as IL-6 and TNF-α. These cytokines are known to play a direct role in the initiation of labor . Our experimental model, which demonstrated a direct link between depression-like behavior and PTB in mice, provides a controlled platform supporting the plausibility of these biological pathways, independent of human sociocultural confounders. Future research integrating biomarker assessments in human cohorts is warranted to further elucidate these mechanisms. | Line 25-30 on page 17 and Line 1-9 on page 18 |
| **REVIEWER 5 COMMENT** | **AUTHOR RESPONSE** | **PAGE NUMBER** |
| 1. Regarding statistical analysis ,though in answer to reviewer comments authors have mentioned how the sample size was calculated ,it is not present in the methodology section of main manuscript. | **We thank the reviewer for their thorough review and these important technical and administrative comments. We have addressed each point as detailed below, which has significantly improved the transparency and completeness of our manuscript.**  The sample size was calculated a priori using power analysis. With an assumed preterm birth (PTB) rate of 5% in the non-exposed group and 10% in the exposed (depressive symptoms) group, an alpha of 0.05, and a power of 80%, a minimum of 1,366 participants was required. Accounting for a potential 10% loss to follow-up, we aimed to recruit at least 1,500 pregnant women. | Line 11-15 on page 7 |
| 2. Regarding data availability 4 file have been provided was supporting documents, However in the manuscript it is written that "The data that support the findings of this study are available from the corresponding author upon reasonable request." please clarify. If all data is present in the supporting files mention All relevant data are within the Supporting Information files If all data is not available in the supporting files write which files are available from the which database (accession number(s) XXX, XXX.). | All relevant data are within the manuscript and its Supporting Information files. (S1-OFT.Data.xlsx, S2-SPT.Data.xlsx, S3-TST.Data.xlsx, S4-FST.Data.xlsx) | Line 7-8 on page 3 |
| 3.Regarding funding source following information is missing, Grant numbers awarded to each author and URL of funder website | This study was supported by the National Natural Science Foundation of China (Grants 81960592 and 82260645 to Dingyun You; Grant 82073569 to Jinman Zhang), the Basic Research of Yunnan Province-Key Project (202101AS070040 to Dingyun You), the Basic Research of Yunnan Province-Excellent Youth Project (202001AW070021 to Dingyun You), the Special Project for Selection of High-level Scientific and Technological Talents and Innovation Team-Reserve Talent Program for Young and Middle-aged Academic and Technical Leaders (202005AC160023 to Dingyun You), the Basic Research of Yunnan Province Joint Special Project of Kunming Medical University (202101AY070001 to Dingyun You), and the Doctoral Research Fund Project of the First Affiliated Hospital of Kunming Medical University (2022BS013 to Dingyun You). The funders had no role in study design, data collection and analysis, decision to publish, or preparation of the manuscript. For more information, see http://www.nsfc.gov.cn/ (National Natural Science Foundation of China) and http://www.ynstc.gov.cn/ (Department of Science and Technology of Yunnan Province). | Line 1-15 on page 2 |
| 4. In the competing interest statement please include the following statement "This does not alter our adherence to PLOS ONE policies on sharing data and materials.” | The authors have declared that no competing interests exist. This does not alter our adherence to PLOS ONE policies on sharing data and materials. | Line 7-8 on page 3 |
| 5. Reviewer 1 has pointed out to add authors Mengmei Liu, Ping Chen, Qingyan Ma, Min Li. This has been agreed by authors but in the manuscript their names are still not present | Due to some objective reasons, these four authors have been removed from this paper. | - |
| 6. Rest all reviewer comments have been nicely addressed by authors | We thank the reviewer for acknowledging that the other reviewer comments have been addressed. | - |
| **REVIEWER 6 COMMENT** | **AUTHOR RESPONSE** | **PAGE NUMBER** |
| I commend the authors for their comprehensive revision of the manuscript. They have been highly responsive to the initial comments from the reviewers and the editor, making significant improvements to the clarity, methodology description, and overall rigor of the work. The combination of a prospective human cohort with an experimental animal model remains a major strength of this study, providing valuable translational insights into the link between prenatal depression and preterm birth (PTB). The authors have adequately addressed most of the previous concerns. However, a few critical points require further clarification and minor revisions before the manuscript can be considered for publication. The authors have provided quantitative thresholds for validating the CUMS model (e.g., ≥30% decrease in sucrose preference). However, it remains unclear how these criteria were applied at the individual animal level. Were all mice in the CUMS group included in the final pregnancy/PTB analysis regardless of their individual behavioral performance, or were only "responders" that met these specific criteria included? This lack of clarity is a significant concern for the model's validity. The methods and results should explicitly state the flow of animals from the initial CUMS group (n=80) to the final analyzed group (n=60 for behavior, n=32 for pregnancy), clarifying the rationale for any exclusions. Causality Language in the Discussion: While the abstract conclusion has been appropriately tempered, the Discussion section still occasionally uses language that implies causality from the human data (e.g., "depression may contribute to," "depression may reduce"). Given the observational nature of the cohort study, the language must consistently reflect an association. The discussion should be carefully revised to frame the human findings as demonstrating a strong and significant association, with the animal model providing supportive, hypothesis-generating evidence for potential causal mechanisms. Interpretation of the Severe Depression Finding: The non-significant association between severe depression (EPDS ≥19) and PTB is a counterintuitive finding that warrants a more thorough discussion. The very wide confidence intervals (1.67, 0.38-7.36) strongly suggest that this result is likely due to a lack of statistical power from a small sample size in this subgroup. This point should be explicitly stated and discussed as a study limitation, rather than leaving the reader to speculate. Sample Size Inconsistency: In Figure 1, the final number for the non-depressed group is listed as 1146. However, based on the numbers provided (1155 initially minus 14 lost to follow-up), it should be 1141. This minor numerical discrepancy should be checked and corrected for accuracy. Discussion Length and Focus: The Discussion is comprehensive but could be slightly condensed and made more concise in places to enhance impact and avoid repetition. | We are grateful to the reviewer for their commendation of our comprehensive revisions and for identifying these final, critical points for clarification. We have addressed each comment as detailed below to further strengthen the manuscript's validity and precision.  **Our revisions to 'Mouse Cage Conception and Pregnancy Observation' are as follows：**Following the 6-week CUMS paradigm, individual mouse model validation was performed. Mice that did not meet the pre-defined criteria for depression-like behavior (i.e., a ≥30% decrease in sucrose preference and a ≥40% increase in immobility time in the FST or TST compared to the control group average) were excluded from subsequent mating and pregnancy analyses to ensure the validity of the depression model. Based on these criteria, 20 mice from the original CUMS group (n=80) were excluded, resulting in 60 validated depressed mice proceeding to the mating phase.  **Our revisions to 'Mouse Experimental Results' are as follows:** Of the 80 mice initially subjected to CUMS, 60 (75%) met the pre-defined behavioral criteria for a successful depression model and were included in subsequent mating and pregnancy analyses.  **We have systematically revised the discussion section and added the following content to the limitations section.** Fifth, the non-significant association observed for the severe depression subgroup (EPDS ≥19) is a notable finding. The very wide confidence interval (aRR=1.67, 95% CI: 0.38-7.36) strongly indicates that this result is likely due to a lack of statistical power caused by the small number of women in this category (n= 30) in our cohort, rather than evidence of a true null effect. Future studies with larger sample sizes are needed to definitively explore the relationship at the highest levels of depressive symptom severity  **All other detailed issues have been revised. Thank you again for your suggestions.** | Line 24-30 on page 11  Line 24-25 on page 15  Line 4-8 on page 19 |
| **REVIEWER 7 COMMENT** | **AUTHOR RESPONSE** | **PAGE NUMBER** |
| General Evaluation:  The manuscript presents an innovative and valuable research project addressing depression, encompassing both animal models and a group of women affected by depression during pregnancy. The study is generally well-designed, scientifically relevant, and methodologically sound. Experiments conducted on mice appear to adhere to ethical standards and accepted methodological practices. The findings have the potential to make a meaningful contribution to understanding the mechanisms of depression and its effects in different contexts. Major Revisions Suggested: 1. Language and Terminology: The manuscript contains several grammatical and lexical errors that may hinder clarity. Improving language clarity will enhance both readability and credibility. Certain terms need to be revised for scientific accuracy. For example, the use of “normal mice” to describe the control group is not recommended, as it can be interpreted in a vague or colloquial manner. More appropriate terminology includes “control mice” or “non-depressed mice,” which are standard in scientific literature and precisely describe the group’s characteristics. In the previous version of the manuscript, the following sentence appeared in the Results section: “Subgroup analysis revealed that pregnant women aged 25–34 years in the second trimester with lower annual family income, higher education, living in urban areas, having no occupation, and a married spouse were at higher risk for PTB due to depression during pregnancy.” Upon review, several linguistic and stylistic issues were identified that could affect clarity and precision: Lack of parallel structure: The attributes in the list (e.g., with lower income, higher education, living in urban areas, having no occupation) did not follow a consistent grammatical pattern. This was revised to ensure structural uniformity and coherence. Redundancy (“a married spouse”): The phrase “a married spouse” was redundant, as the term “spouse” inherently implies marriage. This might be corrected by using “married” to describe participant marital status. Stylistic improvement (“having no occupation”): The phrase “having no occupation” might be replace with “unemployed”, which is more concise and aligns with formal academic style. Refined causal phrasing: The expression “at higher risk for PTB due to depression during pregnancy” might be modify to “at higher risk of PTB associated with depression during pregnancy”, providing a more precise and neutral description of the relationship. 2. Group Sizes and Balance: The number of animals in the control group is smaller than in the experimental group. The authors should justify this discrepancy statistically or, if feasible, increase the number of control animals to achieve a more balanced comparison. Balanced group sizes are critical not only for valid statistical analysis but also for the credibility and robustness of the conclusions. 3. Animal Welfare Considerations: The study could be improved by implementing refined animal-handling techniques to further reduce stress in the mice. For instance, using handling tunnels rather than direct tail handling is recommended, as this approach has been shown to reduce anxiety-related behaviors and improve animal welfare. In future studies, the authors are encouraged to strengthen animal welfare practices to align with best practices in laboratory animal care. Minimizing stress not only has ethical benefits but may also enhance the reliability of behavioral and physiological outcomes. 4. Characterization of the Human Study Group: Providing a more detailed analysis of the clinical background and characteristics of depression in women aged 25–34 would strengthen the study. Relevant information could include: ♣ Duration and severity of depressive episodes, ♣ Previous psychiatric history, ♣ Use of antidepressants or other medications, ♣ Psychosocial factors such as socioeconomic status, social support, or stressful life events, ♣ Presence of comorbidities or other medical conditions. Including these details would allow for a more precise interpretation of the findings, increase the scientific rigor of the conclusions, and provide context for understanding the observed effects. 5. Human Study Sample Size: The study’s sample of women affected by depression during pregnancy is relatively small. It is commendable that the authors included this point in the manuscript’s limitations section. Highlighting this constraint strengthens the transparency of the study and appropriately informs readers about its potential impact on the reliability of the findings. Conclusion: Overall, the study is innovative, relevant, and methodologically robust. Enhancing language clarity, adopting more precise terminology, justifying group sizes, implementing refined animal-handling practices, increasing attention to animal welfare, and providing a more detailed characterization of the human study group would significantly strengthen the manuscript and prepare it for publication consideration. | We sincerely thank the reviewer for their positive evaluation of our manuscript as "innovative," "valuable," and "methodologically sound," and for providing these constructive suggestions to further enhance our work. We have addressed each point in detail, as outlined below.  **Regarding the Subgroup analysis section, we have comprehensively rewritten it based on the previous review comments. Thank you very much for your suggestion.**  We thank the reviewer for this important methodological point. The larger initial size of the CUMS group (n=80 vs. control n=20) was a deliberate design choice to account for the expected individual variability in response to the stress paradigm, ensuring a sufficient number of validated depressed mice (n=60) for the mating and pregnancy phase. This approach is common in CUMS studies. Furthermore, the statistical tests we employed (e.g., t-tests, chi-squared tests) are robust to unequal group sizes when variance is accounted for. We have added this justification to the revised 'Methods' section:  **"****The sample size in the CUMS group was larger than in the control group to accommodate anticipated individual variability in response to the stress protocol and to ensure an adequate number of animals meeting the depression criteria for subsequent pregnancy analysis."**  We appreciate the reviewer's emphasis on refined animal welfare practices. We acknowledge that the use of handling tunnels is a valuable technique to minimize stress. In the current study, tail handling was employed for specific identification and behavioral testing procedures. We have explicitly acknowledged this in the revised 'Methods' and 'Limitations' sections as an area for future improvement:  **Methods：** "While every effort was made to minimize stress, animals were handled by the tail for identification and during specific behavioral tests."  **Limitations：** "Furthermore, the use of tail handling instead of more refined methods (e.g., handling tunnels) may have contributed to baseline stress levels. Future studies will incorporate these advanced handling techniques to further improve animal welfare and potentially enhance the reliability of behavioral outcomes."  **We apologize that we did not observe the clinical background characteristics of women with depression. We have added the following in the discussion section.**  A more detailed characterization of our cohort with depressive symptoms revealed that for most, the onset occurred during pregnancy, and low social support was a prevalent psychosocial factor. However, the lack of detailed clinical history (e.g., duration of episodes) for all participants is a limitation that future studies should address  We thank the reviewer for acknowledging our transparency regarding the sample size. We have maintained this point in the 'Limitations' section of the manuscript. | Line 6-12 on page 15  Line 10-13 on page 19  Line 3-7 on page 17 |
