# Supplementary material for: Association between depression during pregnancy and preterm birth: Results from population cohorts and mouse experimental models
Source: PLoS One. 2026 Jan 29;21(1):e0341449. doi: 10.1371/journal.pone.0341449 (PMC12854446; doi:10.1371/journal.pone.0341449)
Supplement: Attachment — Submitted filename: Response to Reviewers.docx [file pone.0341449.s013.docx]

# Author response to academic editor and reviewers

MANUSCRIPT ID: PONE-D-24-60622

MANUSCRIPT TITLE: Association between depression during pregnancy and preterm birth: results from population cohorts and mouse experimental models

| **academic editor** | **AUTHOR RESPONSE** | **PAGE NUMBER** |
| --- | --- | --- |
| 1.The authors have not adequately addressed this comment in the revised manuscript. Please provide a clear response and incorporate the necessary revisions. Language and Terminology:  The manuscript contains several grammatical and lexical errors that may hinder clarity.  Improving language clarity will enhance both readability and credibility.  Certain terms need to be revised for scientific accuracy. For example, the use of  “normal mice” to describe the control group is not recommended, as it can be  interpreted in a vague or colloquial manner. More appropriate terminology includes  “control mice” or “non-depressed mice,” which are standard in scientific literature and  precisely describe the group’s characteristics.  2.In the previous version of the manuscript, the following sentence appeared in the  Results section:  “Subgroup analysis revealed that pregnant women aged 25–34 years in the second  trimester with lower annual family income, higher education, living in urban areas,  having no occupation, and a married spouse were at higher risk for PTB due to  depression during pregnancy.”  Upon review, several linguistic and stylistic issues were identified that could affect  clarity and precision:  Lack of parallel structure:  The attributes in the list (e.g., with lower income, higher education, living in urban  areas, having no occupation) did not follow a consistent grammatical pattern. This was  revised to ensure structural uniformity and coherence.  Redundancy (“a married spouse”):  The phrase “a married spouse” was redundant, as the term “spouse” inherently implies  marriage. This might be corrected by using “married” to describe participant marital  status.  Stylistic improvement (“having no occupation”):  The phrase “having no occupation” might be replace with “unemployed”, which is more  concise and aligns with formal academic style.  Refined causal phrasing:  The expression “at higher risk for PTB due to depression during pregnancy” might be  modify to “at higher risk of PTB associated with depression during pregnancy”,  providing a more precise and neutral description of the relationship. | We sincerely thank the reviewer for pointing out the issues regarding language clarity and scientific terminology. We agree that precise language is crucial for the credibility and readability of the manuscript. We have thoroughly revised the manuscript to address these concerns:  Terminology for Animal Groups: As suggested, we have replaced the non-specific term “normal mice” throughout the manuscript with the more precise and standard terms “control mice” or “non-stressed control mice”. This change has been made in the Abstract, Results, and Discussion sections to accurately reflect the experimental design.  Language Clarity and Grammar: We have carefully proofread the entire manuscript to correct grammatical errors, improve sentence structure, and enhance overall clarity. Specific revisions include:  Correcting subject-verb agreement (e.g., “A questionnaire was employed”).  Refining awkward or imprecise phrases for better flow (e.g., rephrasing “comparison studies” to “A comparison of existing evidence”).  Replacing colloquial or inaccurate terms with scientific language (e.g., replacing “depressed patients” with “women with elevated depressive symptoms”).  Removing an out-of-context sentence in the Discussion that lacked prior methodological support.  We believe these comprehensive edits have significantly improved the linguistic quality and scientific rigor of the manuscript, addressing the reviewer’s valid concerns.  We thank the reviewer for this meticulous and constructive feedback on the linguistic style of our results presentation. We fully agree that precision and clarity in wording are paramount.  We have revised the problematic sentence in the Results section to address all the raised points:  Parallel Structure: We have rephrased the list of characteristics to ensure grammatical consistency and structural coherence.  Redundancy & Style: We have eliminated the redundancy ("married spouse") and replaced the phrase "having no occupation" with the more concise and standard term "unemployed".  Causal Phrasing: We have replaced "due to" with "associated with" to more accurately describe the observed relationship, in line with the observational nature of our study.  The revised text now reads:  Pre-specified subgroup analyses were performed to assess the consistency of the association between depressive symptoms and PTB across different population strata (**Figure 3**). The increased risk of PTB associated with depressive symptoms was observed consistently across most subgroups. The point estimates were highest for women who were unemployed, resided in urban areas, or had a college/bachelor's degree or above.  We believe this revision significantly improves the clarity, precision, and academic tone of the manuscript. | Line 23,26,27 on page 4.  Line 13-16 on page 5.  Line 10-16 on page 15. |
| 3. Animal Welfare Considerations:  In the section concerning animal welfare, it is important to emphasize the use of low-stress handling methods (e.g., using tunnels) outside of controlled procedures that may require a specific approach and tail handling. Please take this into consideration, as it may improve the quality of the results obtained. | We thank the reviewer for raising this important point regarding animal welfare and its impact on research quality. We fully agree that distinguishing between procedure-required handling and routine low-stress management is crucial.  To address this, we have added a dedicated subsection titled “Animal Handling and Welfare Considerations” within the Methods section (following the description of behavioral tests). In this new paragraph, we explicitly state:  That tail handling was limited to specific experimental protocols where it was an integral part of the test (e.g., the Tail Suspension Test).  That for all other routine husbandry and handling activities (e.g., transfers, identification), we prioritized the use of low-stress methods, such as handling tunnels or cupping, to minimize baseline stress.  This revision clarifies our comprehensive approach to animal welfare, aligns with best practices, and acknowledges the reviewer’s insight that such practices can contribute to the reliability of behavioral outcomes.  We believe this addition strengthens the methodological rigor of our manuscript. | Line 14-22 on page 10. |
| 4. Characterization of the Human Study Group:More detailed investigation of additional factors that may strongly influence depression, such as environmental conditions, social interactions, or genetic background, would help avoid false-positive or false-negative results. This is particularly important when the experimental group is small, as limited sample sizes can increase variability and reduce statistical power. Careful control and reporting of these factors would improve the reliability and interpretability of the findings. | We sincerely thank the reviewer for raising this critical methodological point. We fully agree that unmeasured or insufficiently characterized confounding factors—such as detailed social environment, life history, and genetic background—can influence both depression risk and pregnancy outcomes, potentially affecting the validity of observed associations, especially in studies with moderate sample sizes.  In direct response to this comment, we have added a new, dedicated paragraph to the “Strengths and Limitations” section of the Discussion (now the second point). In this paragraph, we explicitly:  Acknowledge that our study did not capture key psychosocial and biological factors (e.g., social support, trauma history, genetic risk).  State that this represents a source of potential residual confounding.  Note the relevance of this limitation to our sample size considerations.  Propose that future research with larger cohorts incorporate more comprehensive assessments to address this gap.  We have also re-ordered the points in the Limitations section for better logical flow. We believe this addition significantly improves the transparency and intellectual honesty of our manuscript, providing readers with a clearer framework for interpreting our findings and identifying priorities for future research. | Line 15-19 on page 19. |
